# Neuropsychiatric consequences of Covid 19- CASE REPORT

**DOI:** 10.1192/j.eurpsy.2023.1717

**Published:** 2023-07-19

**Authors:** U. V. Gugleta, S. Tošić-Golubović, O. Žikić, V. Slavković, M. Petkovic

**Affiliations:** 1 Psychiatry clinic, Psychiatry Clinic, University Clinical Centre Nis, Serbia; 2 Psychiatry clinic, Psychiatry Clinic, University Clinical Centre Nis, Nis, Serbia; 3 Center for mental health protection, University Clinical Center Niš, Niš, Serbia, Centar for mental health protection UCC Nis; 4 Faculty of medicine, department of Physiology, Faculty of medicine Nis, Nis, Serbia

## Abstract

**Introduction:**

SARS-CoV-2 is a virus with a multisystem effect, and it can cause numerous neuropsychiatric disorders, both in the acute phase of infection and in the period after the disease has passed. According to Nalbandian et al. (2021), post-acute COVID-19 syndrome is a condition of persistent symptoms and/or delayed long-term complications caused by SARS-CoV-2 infection lasting longer than four weeks after the onset of symptoms.

**Objectives:**

To indicate the possible role of the SARS-COV 2 virus in the development of long-term neuropsychiatric and cognitive consequences of COVID-19.

**Methods:**

We undertook a search of the available medical literature in the period after 2020 with the keywords COVID 19 and neuropsychiatric complications

**Results: Case report:** Female patient, 40 years old, unemployed, married, mother of two children. She was admitted for the first hospital treatment at the Psychiatry Clinic of the UCC of Niš due to psychological disturbances in the form of experiencing her own body changes and changes in the environment, moodiness, anxiety, the conviction that she is suffering from incurable diseases, the experience of being centered and existentially threatened, insomnia. In 2020, one month after the recovery from COVID-19, she was treated at the Neurology Clinic of the UCC of Niš for a crisis of consciousness, diagnosis at discharge: encephalitis, encephalopathy. At the end of the treatment, cognitive-mnestic deficits remain. In April 2021, after reinfection with the SARS-COV2 virus, a depressive-interpretive syndrome developed, which is the reason for the current hospitalization. Depersonalization and derealization phenomena, time disorientation, hypochondrial delusions, ideas of self-accusation, cenesthetic hallucinations, impaired volitional-instinctual dynamism and deficits in cognitive-mnestic functioning are observed during hospitalization. NMR of the endocranium with contrast shows changes in the form of encephalomalacia, porencephaly, which indicates a condition after a cerebrovascular insult. She was treated with low doses of haloperidol (2 mg pd), antidementia and vasoactive therapy, which led to a reduction of psychotic symptoms as well as an initial improvement in cognitive-mnestic functioning.

**Conclusions:**

This case report confirms the neurotoxicity of the SARS-CoV-2 virus and it is in accordance with the available literature. The neuropsychiatric and cognitive complications that accompany COVID-19 are different and have a significant impact on the health of people who recovered from COVID-19. It is necessary for the health system to recognize this problem in time and provide organized neuropsychiatric and cognitive monitoring to patients suffering from COVID-19.

**Disclosure of Interest:**

None Declared

